# Distinct and developmentally regulated activity-dependent plasticity at descending glutamatergic synapses on flexor and extensor motoneurons

**DOI:** 10.1038/srep28522

**Published:** 2016-06-22

**Authors:** Constanze Lenschow, Jean-René Cazalets, Sandrine S. Bertrand

**Affiliations:** 1 INCIA, Université de Bordeaux, CNRS UMR5287, 33076 Bordeaux, France

## Abstract

Activity-dependent synaptic plasticity (ADSP) is paramount to synaptic processing and maturation. However, identifying the ADSP capabilities of the numerous synapses converging onto spinal motoneurons (MNs) remain elusive. Using spinal cord slices from mice at two developmental stages, 1–4 and 8–12 postnatal days (P1–P4; P8–P12), we found that high-frequency stimulation of presumed reticulospinal neuron axons in the ventrolateral funiculus (VLF) induced either an NMDA receptor-dependent-long-term depression (LTD), a short-term depression (STD) or no synaptic modulation in limb MNs. Our study shows that P1–P4 cervical MNs expressed the same plasticity profiles as P8–P12 lumbar MNs rather than P1–P4 lumbar MNs indicating that ADSP expression at VLF-MN synapses is linked to the rostrocaudal development of spinal motor circuitry. Interestingly, we observed that the ADSP expressed at VLF-MN was related to the functional flexor or extensor MN subtype. Moreover, heterosynaptic plasticity was triggered in MNs by VLF axon tetanisation at neighbouring synapses not directly involved in the plasticity induction. ADSP at VLF-MN synapses specify differential integrative synaptic processing by flexor and extensor MNs and could contribute to the maturation of spinal motor circuits and developmental acquisition of weight-bearing locomotion.

In the central nervous system (CNS), chemical synapses can encode their history through activity-dependent synaptic plasticity (ADSP). Such changes in synaptic strength, which have been proposed to serve as the cellular basis of learning and memory, are part of the global function of CNS synapses and play an active role in their maturation and stabilization during development. In the spinal cord, motoneurons (MNs) that must integrate thousands of convergent synaptic inputs undergo major post-natal maturation^1^. While ADSP expressed at synapses onto MNs should be instrumental to both MN integrative capabilities and synaptic maturation, few such data are available for mammalian spinal motor synapses, with knowledge mainly being restricted to spinal sensory afferent-MN connections^2^,^3^,^4^,^5^,^6^,^7^.

At birth, rodent pups are unable to walk due to a still immature control of postural adaptations. With development, a rostrocaudal gradient of postural control is formed, beginning with head and shoulders support during the first postnatal week, followed by an elevation of the pelvic region in the second postnatal week. Then, by postnatal day 10 (P10), pups are capable of raised quadrupedal posture^8^. This rostrocaudal gradient has been correlated with the rostrocaudal invasion of the spinal cord by descending pathways. The reticulospinal system is one major descending pathway involved in triggering locomotion and maintaining postural control. Neurons of the reticular formation contact spinal MNs via the ventrolateral funiculus (VLF) of the spinal cord^9^,^10^,^11^. During rodent spinal cord ontogenesis, the first fibres originating from the reticular formation reach the cervical level by embryonic day (E)13–E14 and invade the lumbar spinal cord just before birth. The first postnatal week is then characterized by a rostrocaudal increase in reticulospinal fibre density in the spinal cord^12^,^13^,^14^,^15^. This postnatal anatomical maturation is paralleled by synaptic latency shortening and an amplitude increase of the monosynaptic component of VLF-evoked responses in MNs^16^.

In this context, the aim of the present study was to decipher the ADSP capabilities of VLF-MN synapses and to assess how spinal cord circuit development impacts on ADSP expression in these connections. For this purpose, the plasticity-inducing effects of physiologically relevant high frequency stimulation applied to VLF axons (VLF-HFS) were explored at VLF-MN synapses in spinal cord slices during the acquisition of postural control in P1–P4 and P8–P12 mouse pups. We find that different forms of ADSP can be expressed following VLF axon tetanisation at VLF-MN synapses. Moreover, we identified a rostrocaudal maturation of ADSP expression in MNs during development and revealed that ADSP expression is a function of their flexor and extensor phenotype.

## Results

**Figure 1:**
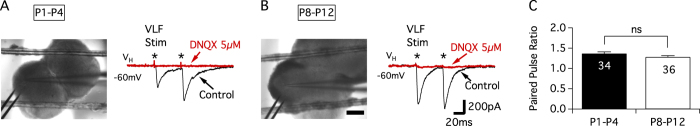
Characterization of VLF-induced EPSCs in P1–P4 and P8–P12 lumbar MNs in mouse spinal cord slices. Representative bright-field images of spinal cord slices (scale bar: 200 μm) and sample traces of EPSCs elicited during paired-pulse stimulations of VLF axons (VLF stim, 50 ms interval, asterisks) in lumbar MNs held at −60 mV (V_H_ −60 mV) of P1–P4 (**A**) and P8–P12 (**B**) mice. In the presence of the AMPA/kainate-receptor antagonist, DNQX (5 μM, red traces), control VLF-EPSCs (black traces) were completely suppressed in both age ranges tested (**A,B**). This effect was observed in all MNs tested (n = 8 for both P1–P4 and P8–P12 mice). (**C**) Paired-pulse ratio value (PPR = EPSC2/EPSC1) computed in all P1–P4 and P8–P12 MNs tested in this study. ns: not significantly different, Mann Whitney test, p = 0.22.

Irrespective of age, under baseline conditions of VLF stimulation (0.03 Hz), AMPA/kainate receptor-mediated excitatory postsynaptic currents (VLF-EPSCs) were recorded in the presence of GABAergic and glycinergic receptor antagonists from lumbar motoneurons held at −60 mV in spinal cord slices (Fig. 1A,B). During paired pulse stimulations (at 50 ms intervals), VLF-EPSCs exhibited paired pulse facilitation (PPF) in both age groups tested (Fig. 1A,B). Similar paired pulse ratio (PPR = EPSC2/EPSC1) values were obtained in P1–P4 and P8–P12 MNs (Mann Whitney test, p = 0.22) suggesting no major differences in release probability between the two groups (Fig. 1C).

During locomotion, reticulospinal neurons in cats have been shown to discharge at ~50 Hz^17^,^18^. This frequency is also classically used to evoke VLF-induced fictive locomotion in *in vitro* en bloc spinal cord preparations from new-born rodents^19^. Based on these data, therefore, we chose to assess the plasticity-inducing effects of 50 Hz high frequency stimulations applied in 2 s trains to VLF axons (VLF-HFS).

**Figure 2:**
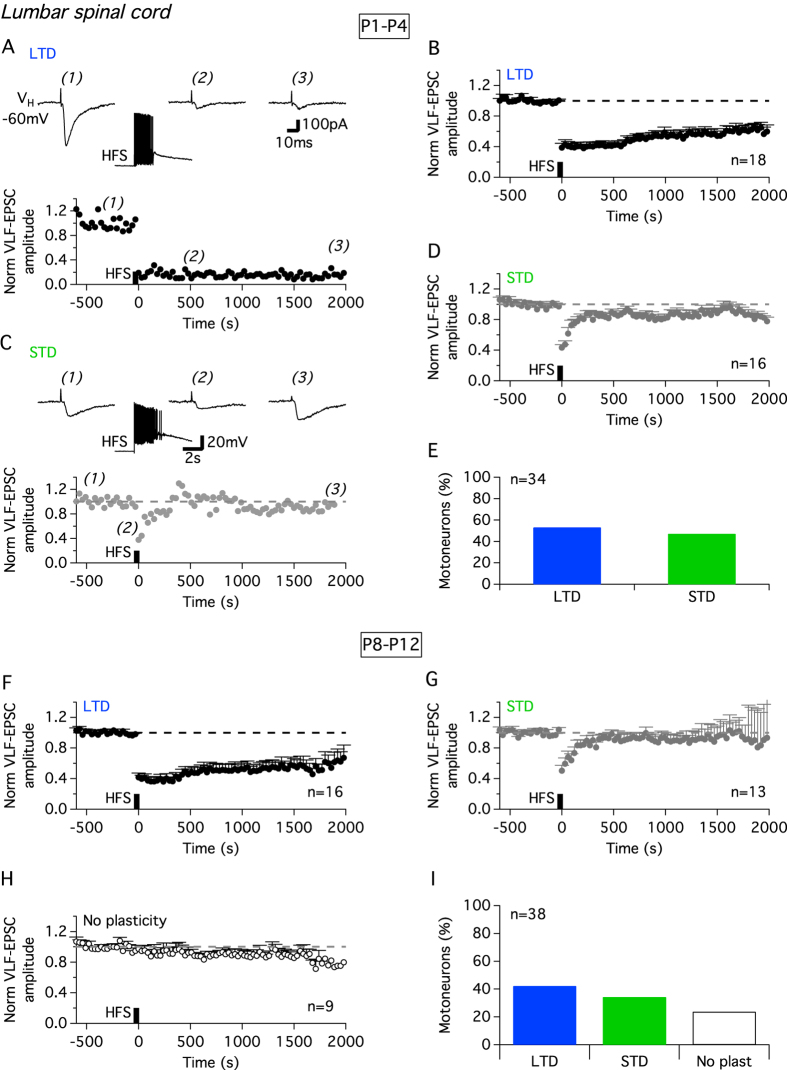
Activity-dependent synaptic plasticity at VLF-lumbar MN synapses of P1–P4 and P8–P12 mice. Representative experiments depicting VLF-HFS (50 Hz, 2 s)-induced LTD (**A**) and STD (**C**) in P1–P4 lumbar MNs. Sample traces of VLF-EPSCs before (1), at different time points after HFS (2 and 3), and during VLF-HFS-induced depolarization (middle trace in **A**,**C**). Pooled data average time courses of normalized VLF-EPSC amplitude in MNs expressing LTD (**B**) or STD (**D**). (**E**) Percentages of different ADSPs expressed by lumbar MNs in P1–P4 mice. Pooled data for LTD (**F**) and STD (**G**) time courses or absence of plasticity (**H**) in P8–P12 lumbar MNs after VLF-HFS. (**I**) Equivalent data presentation as in (**E**) for P8–P12 mice.

As illustrated in Fig. 2A–D, VLF-HFS applied after a 10 min period of stable baseline recording produced a marked depression in VLF-EPSC amplitude in all lumbar MNs tested in P1–P4 mice (n = 34 neurons from 25 animals). This depression either lasted throughout the recording period (more than 30 min) indicating a long-term depression (LTD; Fig. 2A,B), or amplitudes returned to baseline values around 300 s after HFS, consistent with a short-term depression (STD) of VLF-EPSCs (Fig. 2C,D). MNs were categorized into STD-expressing MNs when VLF-EPSC amplitude exceeded 80% of the pre-HFS baseline control value 500 s after HFS. In P1–P4 mice, LTD and STD were equally distributed among the lumbar MNs tested (Fig. 2E). The PPF evoked by two stimuli at an interval of 50 ms was not altered after the induction of LTD (PPR = 1.3 ± 0.05 pre-HFS and 1.02 ± 0.01 30 s post-HFS; n = 17, p = 0.46 Wilcoxon test) suggesting that the expression locus of LTD was postsynaptic. In contrast, the PPR was significantly decreased during STD in lumbar MNs (PPR = 1.4 ± 0.04 pre-HFS and 1 ± 0.09 30 s post-HFS; n = 15, p = 0.0043 Wilcoxon test) indicating a presynaptic locus of STD expression.

To seek for developmental changes, the effects of VLF-HFS were tested during the second postnatal week (P8–P12) in lumbar MNs (Fig. 2F–I; n = 38 neurons from 24 animals). VLF-HFS applied to P8–P12 lumbar MNs led to either LTD (Fig. 2F) or STD (Fig. 2G) of VLF-EPSC amplitudes. However, unlike in P1–P4 animals, VLF-MN synapses devoid of post-VLF-HFS modulation (No plasticity; Fig. 2H) were also observed. The occurrence of these non-plastic synapses following VLF-HFS in P8–P12 animals thus appears to be a feature of this developmental stage at the lumbar level (compare Fig. 2E,I).

The amplitudes of STD and LTD in lumbar MNs tended to remain constant throughout the developmental period investigated. Indeed, no significant difference in the magnitude of the synaptic depression was observed regardless of both the type of plasticity expressed and the developmental stage tested (lumbar P1–P4 LTD: −61 ± 06% of the mean VLF-EPSC baseline amplitude, n = 18; P1–P4 STD: −57 ± 04%; n = 16; P8–P12 LTD-MNs: −58 ± 06%, n = 16; P8–P12 STD-MNs: −50 ± 07%; n = 13; Kruskal Wallis test, p = 0.5).

**Figure 3:**
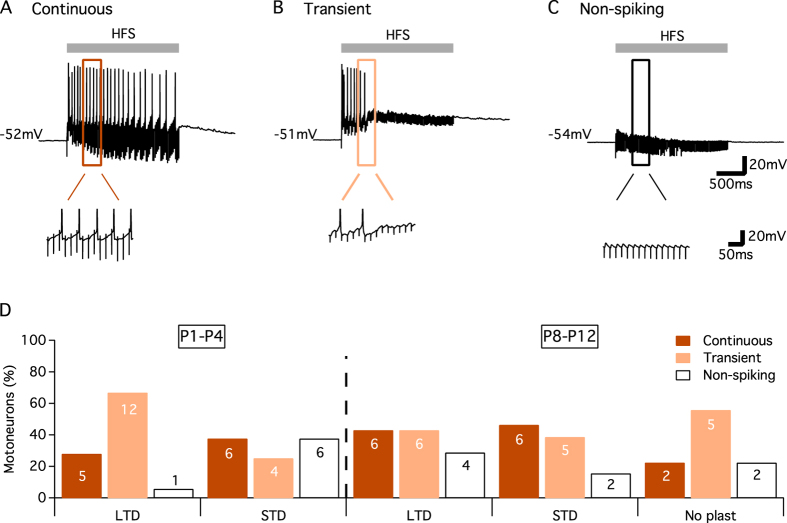
Depolarization profiles of lumbar MNs during HFS. Top traces: representative traces of the three different types of depolarizations recorded in lumbar MNs during VLF-HFS (50 Hz, 2 s). MNs could present a continuous spiking activity (Continuous, **A**), a transient spiking activity (Transient, **B**) or an absence of spike (Non-spiking, **C**). Bottom traces: enlarged view of squares in (**A–C**). (**D**) Repartition plot of the three different patterns of VLF-HFS-induced depolarizations as a function of the ADSP expressed in lumbar MNs of P1–P4 and P8–P12 mice. No significant differences were found between MN types and ages (chi-square test, p = 0.29).

As reported in Table 1, the stimulation intensities used to evoke baseline VLF-EPSCs were not significantly different between MN types and ages (Kruskal Wallis test, p = 0.052). The question then arises as to whether the type of ADSP expressed at VLF-lumbar MNs synapses is linked to the depolarization level reached during HFS? Regardless of age, three different depolarization profiles could be distinguished in lumbar MNs during HFS application (Fig. 3). In most MNs (57 of 72 MNs), HFS triggered large excitatory postsynaptic potentials (EPSPs) that reached the spike threshold. Spiking activity could last for the entire HFS application (Continuous spiking activity; Fig. 3A) or was limited to HFS beginning (Transient spiking activity, Fig. 3B). In the remaining MNs (15 of 75 MNs), HFS-induced EPSPs failed to reach the spike threshold (Non-spiking, Fig. 3C). No significant differences were found between the proportions of continuous, transient or non-spiking MNs displaying different plastic profile (Fig. 3D; chi-square test, p = 0.29). This result indicates that the types of ADSP expressed at VLF-lumbar MNs could not be predicted by the depolarization pattern/amount observed during plasticity induction.

**Figure 4:**
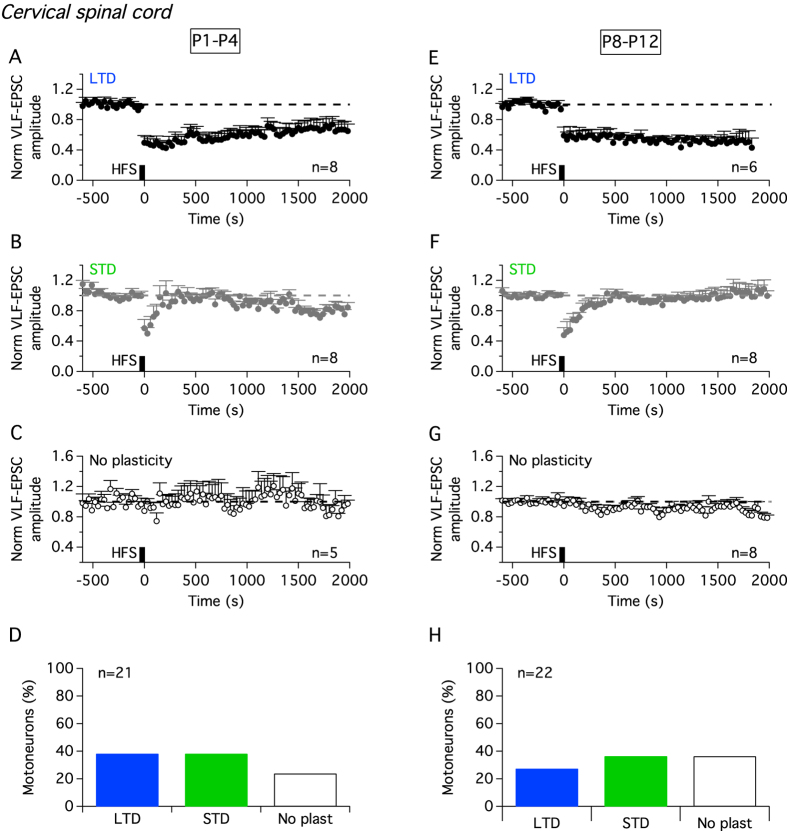
Activity-dependent synaptic plasticity at VLF-cervical MN synapses. Pooled data time course of normalized VLF-EPSC amplitudes in cervical MNs expressing LTD, STD or no plasticity before and after VLF-HFS in spinal cord slices from P1–P4 (**A–C**) and P8–P12 (**E**–**G**) mice. (**D**) Histogram of MN repartition according to ADSP expressed following VLF-HFS in P1–P4 animals. (**H**) Equivalent data presentation as in (**D**) for P8–P12 mice.

During development, there is a rostrocaudal gradient of maturity in the spinal cord correlated with the progressive invasion of supraspinal descending pathways^20^. To examine for possible synaptic plasticity differences related to descending pathway maturation, VLF-HFS was also tested in cervical MNs. Regardless of age, VLF-HFS elicited three different responses at cervical VLF-MN synapses: LTD, STD or no plasticity (Fig. 4A–H). There was no significant difference between the proportions of cervical MNs displaying STD, LTD or no change in P1–P4 and P8–P12 mice (Fig. 4D,H; chi-square test, p = 0.62), and between the plastic profile distributions of P8–P12 cervical and P8–P12 lumbar MNs (Figs 2I and 4H; chi-square test, p = 0.43). P1–P4 cervical MNs, therefore, expressed similar plasticity profiles as P8–P12 lumbar MNs, rather than P1–P4 lumbar MNs.

As previously reported above for lumbar MNs, the amplitudes of STD and LTD reached in cervical MNs were also constant throughout the developmental period investigated (cervical P1–P4 LTD-MNs: −50 ± 09% of mean VLF-EPSC baseline amplitude, n = 8; P1–P4 STD-MNs: −43 ± 11%, n = 8; P8–P12 LTD-MNs: −40 ± 11%, n = 6; P8–P12 STD-MNs: −52 ± 10%, n = 8; Kruskal-Wallis test, p = 0.8). Therefore, altogether these results indicate that ADSP expression at VLF-MN synapses depends on the developmental stage of MNs and exhibits a rostrocaudal gradient of maturation.

**Figure 5:**
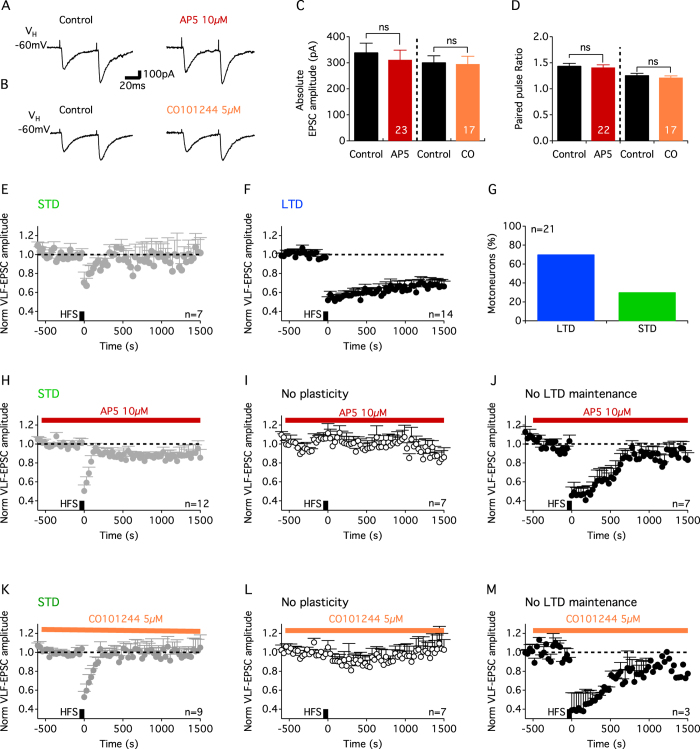
LTD at VLF-lumbar MN synapses in P1–P4 mice is dependent on NMDA receptors containing the GluN2B subunit. Representative traces of VLF-EPSCs recorded during paired-pulse stimulation in control conditions (**A**,**B**) and in the presence of the NMDA receptor antagonist AP5 (**A**) or the potent and selective blocker CO101245 of GluN2B subunit containing NMDA receptors (**B**). Neither baseline VLF-EPSC amplitudes (**C**) nor paired- pulse ratio values (**D**) computed during paired-pulse stimulations were affected by either AP5 or CO101215 (CO). ns: non significantly different, p = 0.052 for EPSC amplitudes and p = 0.6 for PPR values, Wilcoxon test. Time course of STD (**E**) and LTD (**F**) at VLF-MN synapses in spinal cord slices induced by VLF-HFS in control conditions. (**G**). Bar histogram of MN repartition as a function of ADSP expressed at VLF-MNs synapses under control saline conditions. Time courses of normalized VLF-EPSC amplitudes before and after VLF-HFS in the presence of 10 μM AP5, showing an unaffected STD expression (**H**), a blockade of LTD induction (i.e. no plasticity) (**I**) or a blockade of LTD maintenance (**J**). In the latter case, LTD was induced for several minutes but then VLF-EPSC amplitudes returned progressively to baseline during the recording period (**J**). As the time course of this response differed from STD, it was termed ‘no LTD maintenance’. Time courses of normalized VLF-EPSC amplitudes before and after VLF-HFS in the presence of 5 μM CO101244, showing an unaffected STD expression (**K**), a blockade of LTD induction (no plasticity) (**L**) or a blockade of LTD maintenance (**M**).

NMDA receptors (NMDARs) are composed of two obligatory GluN1 subunits and two additional subunits, GluN2 or GluN3, that endow the receptor with specific functional properties. Four different GluN2 subunits have been described: GluN2A, GluN2B, GluN2C and GluN2D. In supraspinal structures, the subunit composition of NMDARs contributes to developmental changes in synaptic plasticity expression with the GluN2B subunit being enriched in immature synapses^21^. By using the NMDAR antagonist, AP5 and the potent and selective GluN2B blocker, CO101245, we found that the activation of NMDARs containing the GluN2B subunit is required for LTD induction and/or maintenance in P1–P4 lumbar MNs (Fig. 5).

MN populations typically exhibit a large degree of heterogeneity and can be categorized into different classes and subtypes^22^,^23^. Besides γMNs that control muscle spindles, MNs that control extrafusal muscle fibres, the αMNs, can be divided into fast and slow subtypes depending on whether they innervate fast twitch or slow twitch muscle fibres, respectively. We then questioned whether the synaptic plasticity expressed at VLF-MN synapses is related to MN functional subtype. Fast and slow MNs are distinguishable by their intrinsic membrane properties: fast MNs are generally relatively large cells characterized by a low input resistance (Rin) and a short afterhyperpolarization (AHP) with small amplitude, while slow MNs are small with a high Rin and long AHP of large amplitude^24^. To assess whether the ADSP type expressed at VLF-MN synapses relies on the fast or slow phenotype of MNs, the values of Rin, AHP amplitude and AHP half decay time (AHP-dt) were compared in MNs classified according to the synaptic plasticity they express following VLF-HFS (Table 1). While P1–P4 MNs expressing different ADSP forms showed some variability in their membrane properties, the absence of significant differences amongst P8–P12 MNs suggests that ADSP expression at VLF-MN synapses is independent of fast or slow phenotype (Table 1). Moreover, neither the baseline amplitude of AMPA-EPSCs nor the baseline PPR computed during paired stimulations were significantly different between MN types and ages (Table 1), indicating that ADSP expression is also not linked to major differences in synaptic release probability between MNs.

**Figure 6:**
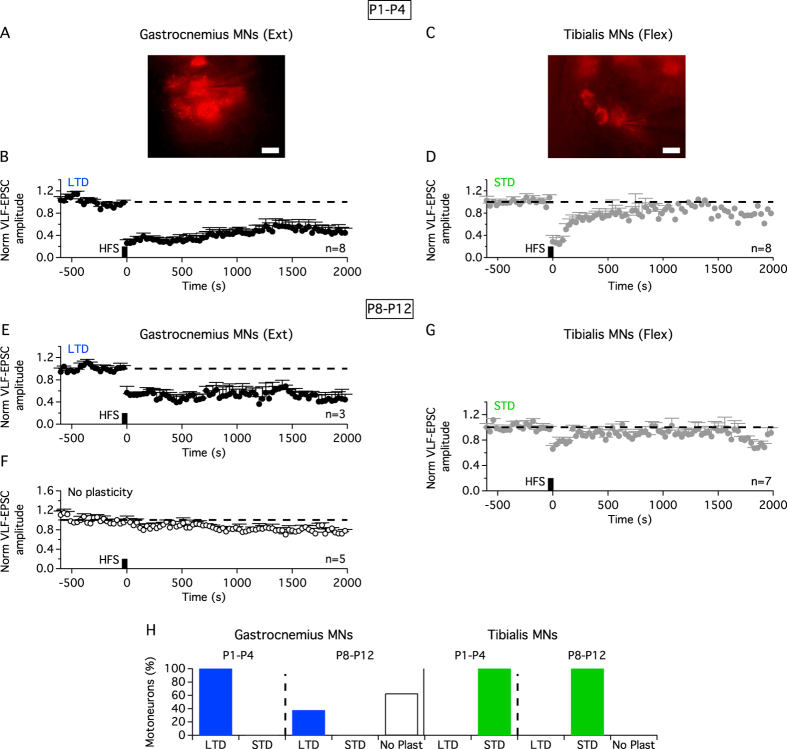
Activity-dependent synaptic plasticity at VLF-lumbar MN synapses is related to hind limb extensor/flexor muscle function. Photomicrographs of retrogradely-labeled MNs of the *Gastrocnemius* (**A**) or *Tibialis Anterior* (**C**) muscles (scale bar: 20 μm). Pooled data time courses of normalized VLF-EPSC amplitudes in *Gastrocnemius* or *Tibialis* MNs before and after VLF-HFS in P1–P4 (**B,D**) and P8–P12 (**E–G**) slices. (**H**) Percentages of *Gastrocnemius* and *Tibialis* MNs according to ADSP type expressed in the two developmental ranges.

MNs innervating the same target muscle are regrouped into motor pools that have been shown to share molecular markers and functionally defined premotor connectivity^25^,^26^. To determine whether motor pool identity underlies the differential ADSP expression at VLF-MN synapses, VLF-HFS effects were compared between MNs innervating the ankle extensor muscle *Gastrocnemius* and the ankle flexor *Tibialis Anterior* muscle (Fig. 6). In P1–P4 mice, all the retrogradely labelled *Gastrocnemius* MNs recorded (Fig. 6A; n = 8) expressed LTD after VLF-HFS (Fig. 6B,H) while all *Tibialis* MNs (Fig. 6C; n = 8) expressed STD (Fig. 6D,H). In P8–P12 animals, while 100% of recorded *Tibialis* MNs still expressed STD after VLF-HFS (Fig. 6G,H), *Gastrocnemius* MNs were divided into LTD and non-plasticity (No Plast)-expressing MNs (Fig. 6E,F,H). These data therefore suggests that the ADSP type expressed at VLF-MN synapses is dependent on the flexor or extensor role of limb MNs.

**Figure 7:**
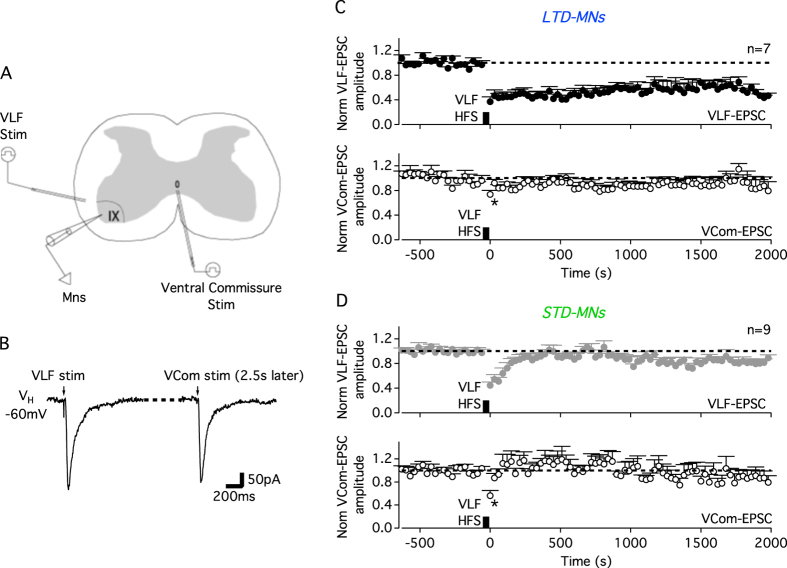
Heterosynaptic plasticity in lumbar MNs. (**A**) Experimental design schematic showing recording (MNs) and stimulating electrode (stim) positions. (**B**) Sample traces of a VLF-induced EPSC followed 2.5 s later by an EPSC evoked by a ventral commissure stimulation (VCom-EPSC). Pooled data time course of normalized VLF-EPSC and VCom-EPSC amplitudes before and after VLF-HFS in MNs expressing LTD (**C**) or STD (**D**) of VLF-EPSCs. *Significantly different from pre-HFS VLF-EPSC value (p ≤ 0.05; Friedman test).

In addition to synapses that are active during plasticity induction, such homosynaptic plasticity could also affect neighbouring inactive synapses and cause heterosynaptic changes in these latter VLF-MN connections^27^. To address such a secondary influence, the heterosynaptic effect of ADSP induction at VLF-MN synapses was investigated on AMPA-EPSCs evoked by electrical stimulation of the ventral commissure in P1–P4 lumbar MNs^28^ (VCom-EPSCs, Fig. 7A,B). In both LTD-expressing MNs expressing (n = 7; Fig. 7C) and STD-expressing MNs (n = 9; Fig. 7D) at VLF-MN synapses, a short-lasting but significant short-time depression (30 s duration) of VCom-EPSC amplitudes was observed in postsynaptic MNs. STD and LTD induction at VLF-MN synapses thus evoked heterosynaptic plasticity in spinal MNs.

## Discussion

The synapses that converge onto spinal MNs are amongst the first connections to be described as expressing frequency-dependent changes in synaptic efficacy^2^. Subsequently, however, ADSP studies on spinal motor networks have been mainly confined to the more readily accessible connections between Ia afferents and MNs, which have been shown to express diverse ADSP phenotypes including post-tetanisation depression, post-activation depression and homeostatic plasticity^7^. The present study now provides novel insights into, and a potential functional relevance of, ADSP expressed at spinal synapses between limb MNs and a major descending pathway, the reticulospinal tract, involved in locomotion initiation and postural regulation. The VLF-MN synapses have been previously shown to exhibit short-term potentiation during 10 Hz train stimulation in the new-born rat^9^. Our data in mice show that VLF-MN synapses express other forms of ADSP such as STD or LTD following physiologically relevant tetanisations^17^. We further demonstrate here that the expression of these plasticity types is developmentally regulated and follows a rostrocaudal gradient of maturation during spinal cord development, including a switch from LTD-expressing to non-plasticity at certain lumbar MN synapses. Intriguingly also, our study reveals that the functional flexor or extensor phenotype of limb MNs determines the type of ADSP expressed at VLF-MN synapses. To the best of our knowledge, this constitutes the first description of differential synaptic integrative properties in flexor and extensor MNs.

In rodents, the acquisition of both locomotor and postural capabilities have been reported to undergo a parallel rostrocaudal evolution during postnatal spinal cord maturation^29^. This rostrocaudal establishment of weight-bearing locomotion (first shoulder then pelvis elevation) has been correlated with MN intrinsic properties maturation and the invasion of the spinal cord by descending neural pathways^8^. Both firing properties and synaptic inputs mature earlier in flexor MNs compared to extensor MNs^16^,^30^. This distinction has been related to lowered gravitational influences and resultant reduced activity of antigravity muscles (i.e., extensor muscles) during the *in utero* life of mammalian foetuses^31^,^32^. Our study reveals that this delay of extensor MN maturation also extends to their integrative synaptic properties, as indicated by the differential changes in ADSP forms. Specifically, a switch from LTD-expressing synapses to non-plastic synapses was found to occur in certain *Gastrocnemius* MNs during early postnatal development, while the plasticity profile of *Tibialis* MNs does not further evolve during spinal motor network maturation. The emergence of non-plastic synapses onto Gastrocnemius MNs thus appears to be an important transitional hallmark of postnatal VLF-MN synapse maturation. Whether ADSP expressed at VLF-MN synapses undergoes further maturational processes after the second postnatal week remains unknown. However, the fact that no differences were observed between P1–P4 and P8–P12 cervical MNs following VLF-HFS suggests that the tripartite plasticity profile (LTD, STD and no plasticity) may persist into adulthood.

The developmental differences we observed between cervical and lumbar MN plasticity profile could rely on the activation of different axonal populations since the spinal VLF tract conveys both descending reticulospinal axons and descending/ascending propriospinal fibres^33^. A contribution of propriospinal and heterogeneous input activation could therefore not be completely excluded in the stimulation data we obtained from spinal cord slices. However, we found that VLF stimulation triggers similar paired pulse and post-HFS plasticity profiles in both cervical and lumbar MNs. In addition, the lack of effect of maturation on ADSP expression at VLF-cervical MN synapses suggest that regardless of the precise source of synaptic inputs activated by VLF stimulations, the ADSP expressed at MN synapses is prone to a rostrocaudal gradient of maturation. These observations together with the well established rostrocaudal development of descending pathways during spinal development^8^ strongly suggests that the results obtained in the present work derived, at least to a large extent, from descending pathway activation. Optogenetic approaches will be required to precisely and specifically activate reticulospinal terminals on MNs in spinal cord slices.

The expression of ADSP has been shown to be developmentally regulated at many different synapses throughout the central nervous system see for exemples: refs 34 and 35. In the spinal cord, the synaptic depression elicited at sensorimotor connections in new-born cat is converted to a post-tetanic potentiation in response to the same conditioning stimulation in adults^36^. Such ADSP conversions have been shown to arise, at least partly, from developmentally regulated changes in NMDAR subunit composition. In the brain, the GluN2B subunit that supports greater Ca^2+^ influx, is highly expressed during the embryonic and early postnatal period, but is progressively replaced by the GluN2A subunit during the second postnatal week^21^. A similar developmental time course has been shown to occur in the ventral motor region of the spinal cord^37^. We found in the present study that NR2B-containing NMDARs are required for the induction/maintenance of LTD in lumbar MNs. This result corroborates with previous findings demonstrating the involvement of NMDARs containing the GluN2B subunit in VLF-MN synaptic transmission in neonatal rats^38^. Therefore, we hypothesize that changes in NR2B/NR2A subunit balance could explain the conversion of a population of LTD expressing VLF-MN synapses into non-plastic synapses during spinal network maturation.

In the lamprey spinal cord, ADSP expressed at synapses in the locomotor central pattern-generating network has been thoroughly investigated^39^. In this model, multiple intracellular recordings of identified pre- and postsynaptic neurons revealed differences in the short-term plasticity of connections from convergent excitatory interneurons onto single MNs^40^, suggesting that ADSP expressed in MNs is determined presynaptically. In contrast, in the cat spinal cord, it has been shown that Ia afferents connecting with a given MN exhibit the same plastic behaviour^41^. Moreover, the magnitude of post-tetanic potentiation (PTP) observed after Ia afferent fiber tetanisation differs between MNs of a same motor pool and is related to both MN input membrane resistance and baseline excitatory postsynaptic potential amplitude^5^. By identifying a link between the flexor/extensor MN phenotype and the plasticity expressed at VLF-MN synapses, our findings therefore strengthen the conclusion that in mammals, MNs themselves are key determinants of the type of ADSP expressed at the synapses they receive. It has been recently shown that the organization of flexor and extensor premotor interneurons differs considerably at the spinal level^25^,^42^. By revealing a MN function-specific plasticity at supraspinal connections, which endows flexor and extensor MNs with different synaptic integrative properties, our study points to different mechanisms of information processing of descending inputs between these two antagonistic motor populations.

Why flexor and extensor MNs express different ADSP forms at VLF-MN synapses remains an open question. Indeed, addressing this issue becomes even more complex when the maturational partition of *Gastrocnemius* MNs into LTD-expressing and non-plastic VLF-MN synapses in older P8–P12 mice is considered. Assessing the role of these different forms of synaptic plasticity, or their absence, during ongoing network activity will be hard to decipher due to the difficulty in isolating specific synapses of interest from all other activated connections. ADSP has been proposed to contribute to the patterning of network activity and regulate motor activity expression^39^,^43^,^44^,^45^. The heterogeneity of synaptic integrative properties between MN pools found in the present study will enable multiple representations of a common presynaptic input, and as suggested by the heterosynaptic plasticity induced in VLF-MN synapses, will also impact on the integration of other converging inputs. Furthermore, the differences observed between limb flexor and extensor MN integrative properties are likely to be related to their distinct antagonistic functions during locomotion, with the specific ADSP repertoire of the two motor pools contributing to the genesis of the precise temporal profile of muscle contractions required for effective locomotion (see for example: ref. 46). An important consequence of ADSP is, indeed, that synapses can act as filters with a wide range of properties. Therefore, the role of the LTD and STD expressed at MN-VLF synapses could be to favour the integration/discrimination of synaptic inputs arising from central pattern generator (CPG) interneurons or sensory inputs, for examples, in MNs during precise temporal windows.

During development, neural network activity and associated ADSP play a major role in the refinement and stabilization of synaptic connections^47^. Correspondingly, the rostrocaudal gradient of ADSP maturation we observed in a descending supraspinal pathway involved in postural control and locomotion initiation could be pivotal to continued motor circuit development and maturation. In any case, ADSP should be paramount to both the acquisition and ongoing production of adult locomotion.

## Methods

### Animals and spinal cord slices preparation

New-born male and female C57Bl/6JRJ mice aged postnatal day 1 (P1) to P4 and P8 to P12 were used in accordance with the guidelines of the French Agriculture and Forestry Ministry for handling animals. The protocol was approved by the local ethics committee of the University of Bordeaux (permit number 5012031A). Mice were anesthetized with 4% isoflurane until noxious reflexes were lost. After decapitation, the spinal cord was dissected out in an ice-cold sucrose-based saline solution containing the following: 2 mM KCl, 0.5 mM CaCl_2_, 7 mM MgCl_2_, 1.15 mM NaH_2_PO_4_, 26 mM NaHCO_3_, 11 mM glucose and 205 mM sucrose. The saline was bubbled with 95% O_2_, 5% CO_2_. Transverse slices (350 μm) of the spinal cervical or lumbar enlargement were cut with a vibroslicer and then transferred to a holding chamber. Slices were allowed to recover in oxygenated aCSF (130 mM NaCl, 3 mM KCl, 2.5 mM CaCl_2_, 1.3 mM MgSO_4_, 0.58 mM NaH_2_PO_4_, 25 mM NaHCO_3_, 10 mM glucose) for at least 1 hour at 30 °C.

### Electrophysiology

Whole-cell voltage- or current-clamp recordings from putative motoneurons (MNs), identified by their relatively large soma size in lamina IX, were made under visual control with a Multiclamp 700B amplifier. Recording glass microelectrodes (4–7 MΩ) were filled with the following: 120 mM K-gluconate, 20 mM KCl, 0.1 mM MgCl_2_, 1 mM EGTA, 10 mM HEPES, 0.1 mM CaCl_2_, 0.1 mM GTP, 0.2 mM cAMP, 0.1 mM leupeptin, 77 mM d-mannitol and 3 mM Na_2_-ATP, with a pH of 7.3. All drugs were obtained from Abcam and Tocris and dissolved in water. All of the experiments were performed at room temperature (~23 °C).

Data acquisition and analysis were performed using Axograph software. Experiments were discarded if series resistance increased more than 20% during a given recording period.

Synaptic responses were evoked by monophasic constant current pulses (100 μs) delivered through a stimulus isolation unit to a bipolar stimulating tungsten electrode (Microprobes, tip separation 75 μm, stimulation intensities ranging from 10 to 60 μA). Stimulating electrodes placed in the ventrolateral quadrant and the ventral commissure of the cord slice were used to stimulate axons of the ventrolateral funiculus (VLF) or commissural axons, respectively. Polysynaptic transmission was decreased using a high cation solution containing 7.5 mM CaCl_2_ and 8 mM MgSO_4_^48^. Throughout recording episodes, GABAergic and glycinergic inputs were blocked with gabazine and strychnine (1 μM each), respectively^49^,^50^. Excitatory postsynaptic currents (EPSCs) were recorded from MNs held at −60 mV in voltage clamp mode. After a 10 min period of stable VLF stimulation (0.03 Hz) at stimulation intensity set to induce sharp EPSCs with fixed latency, a 50 Hz high frequency stimulation (HFS) was applied for 2 s to VLF axons (VLF-HFS) in the spinal cord slices. During VLF-HFS, the intensity of the VLF stimulation was doubled compared to the baseline condition for eliciting EPSCs, and MNs were held in current-clamp mode to allow normal depolarization and firing. VLF-EPSC amplitudes were expressed as values normalized to mean control pre-HFS VLF-EPSC amplitude values.

The input resistance of MNs (Rin) was determined from the slope of the voltage-current curve within the linear portion of current traces. AHP parameters were measured after single action potential evoked by short depolarizing current steps (7 ms, 0.25 nA) in current clamp conditions in MNs held at −60 mV by injection of bias current.

To record specifically from identified MNs innervating the *Gastrocnemius* or the *Tibialis Anterior* muscles, a crystal of cholera toxin β-subunit conjugated to AlexaFluor 594 was inserted into the muscle of interest of the right hind limb with an insect pin 20–24 h before slice preparation procedure in anesthetized mouse pups.

### Statistical analysis

Statistical analyses of raw data were conducted using GraphPad Prism software. Due to some relatively small samples, we used non-parametric tests for all analyses performed. In the text and figures, all data are expressed as means ± SEM. Asterisks in the figures and tables indicate statistical significances (p < 0.05). Wilcoxon, Mann-Whitney, Friedman and Kruskal-Wallis tests with Dunn’s multiple comparisons post-hoc tests were used where applicable. Chi-square tests were used to compare MN proportions displaying STD, LTD or no change at the different spinal levels and ages tested.

## Additional Information

**How to cite this article**: Lenschow, C. *et al*. Distinct and developmentally regulated activity-dependent plasticity at descending glutamatergic synapses on flexor and extensor motoneurons. *Sci. Rep.* **6**, 28522; doi: 10.1038/srep28522 (2016).

## Figures and Tables

**Table 1:**
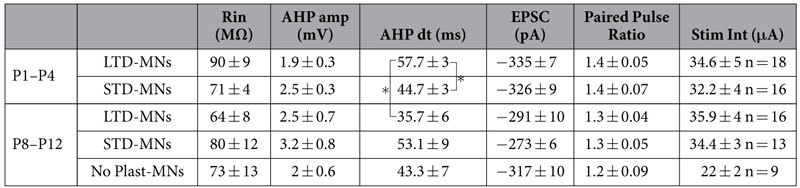
Lumbar motoneuron input membrane resistance (Rin), spike afterhyperpolarization amplitude (AHP amp), AHP half decay time (AHP dt), baseline EPSC amplitude, baseline paired-pulse ratio value (EPSC_2_/EPSC_1_) and baseline VLF stimulation intensity (Stim Int) as a function of ADSP expressed at VLF-MN synapses and mouse age range.

LTD-MNs: LTD-expressing MNs, STD-MNs: STD-expressing MNs, No Plast-MNs: MNs expressing no plasticity, P: postnatal day, n: number of MNs tested. *Significantly different (p ≤ 0.05; Kruskal-Wallis test).
